# A Cost Analysis of the Jan Aushadhi Scheme in India

**DOI:** 10.15171/ijhpm.2017.02

**Published:** 2017-01-17

**Authors:** Kanchan Mukherjee

**Affiliations:** Centre for Health Policy, Planning and Management, School of Health Systems Studies, Tata Institute of Social Sciences (TISS), Mumbai, India.

**Keywords:** Generic Medicines, Branded Medicines, Jan Aushadhi Scheme (JAS), India

## Abstract

Medicines constitute a substantial proportion of out-of-pocket (OOP) expenses in Indian households. In order to address this issue, the Government of India launched the Jan Aushadhi (Medicine for the Masses) Scheme (JAS) to provide cheap generic medicines to the patients (http://janaushadhi.gov.in/about_jan_aushadhi.html). These medicines are provided through the Jan Aushadhi stores established across the country. The objective of this study was to do a quick assessment for policy-makers regarding the objective of the JAS. Implications on cost savings for patients and policy implications of the scheme were analyzed. Secondary data sources were used to obtain prices of medicines under the JAS and prices of branded medicines of the same formulations. A cost analysis design was used. There are substantial differences between the JAS price and the cheapest branded medicine available in the market. However, not all JAS prices are lower than branded medicines. For example, the cheapest branded cefuroxime axetil (500 mg) (antibiotic) in the market is almost three times cheaper than its JAS price. Hence, there are cheaper brands available for some commonly prescribed medicines. From the policy perspective, it raises serious questions regarding the pricing of medicines in the JAS and its overarching goal. Since patients are dependent on physicians for medicine prescriptions and have little knowledge of the price variations among branded and generic medicines, the JAS may not provide the cheapest alternative for the patients. Hence, the government should urgently review the JAS prices to achieve its goal of providing low-cost affordable medicines.

## Background

The trend of using medications to treat diseases has been increasing worldwide since the acceptance of modern medicine as a scientific and reliable mode of treatment. Drugs and medicines form a substantial portion of out-of-pocket (OOP) spending on health among households in India and have become a major source of catastrophic expenditure for both outpatient services as well as inpatient (hospitalization) charges.^1^ Private OOP expenditure by the low income households on medicines is responsible for pushing families into poverty.

The extent of OOP expenditure is so high that some affected patients and households have to take loans to buy medicines. This meant that the expenditure on medicines was responsible for pushing the rural and urban poor into debt. A study over a period of four weeks showed that 41%-56% of households surveyed in low- and middle-income countries had solely spent their health expenses on medicines.^2^ Another study from three north Indian states on burden of medicines showed that the reason for debt was medical expenditure in 25% of the urban households and 19% of rural households,^3^ which suggested that the poor were approaching lenders for borrowing money or selling their assets in order to meet the healthcare expenses. In India, most patients who seek treatment for acute and chronic diseases face a major crisis for accessing essential medicines.^4^ In such situations, it is critical to have alternatives to decrease the OOP expenditure due to medicines.

An ideal solution would be free distribution of medicines; but its financial feasibility for the union government is very poor in India. The Indian government spends just over 1% of the gross domestic product (GDP) on healthcare and repeated union budgets have shown that its nearly impossible to improve the funding to healthcare. Also, neoliberal policies favoring market mechanisms with decreased role of the state in healthcare are being envisaged. Hence, another alternative would be to involve the private sector to decrease the costs of the medicines or distribute them freely, which is impossible due to the profit motivations of this sector. In a situation where the government would not spend more on healthcare and the patient cannot avoid private healthcare services, an important alternative to decrease the patients’ OOP expenditure is through the use of generic medicines. Generic medicines are usually cheaper than branded medicines and are useful in decreasing the expenditure on medicines.^2^ Mass production of generic anti-retroviral medicines saved the lives of millions of HIV/AIDS patients by making the medicines more accessible and affordable in the low- and middle-income countries.^5^ Countries that intended to curtail their expenses and save the citizens from OOP expenditure on medicines adopted policies in favour of generic medicine.^6^

To make generic medicines available to the population, the Department of Pharmaceuticals, Government of India launched the Jan Aushadhi Scheme (JAS) (medicine for the masses) in 2008. Under the scheme, Jan Aushadhi generic drug stores were opened across the country to provide low-cost generic medicines to the population. However, many of these stores are currently non-functional or have closed for various reasons like poor support from state governments, poor supply chain management, non-prescription of generic medicines by the doctors, lack of awareness, etc. Recognizing the weakness in the scheme’s implementation, the government is planning to re-launch its pharmacy chain, Jan Aushadhi stores, to sell generic medicines ranging from antibiotics, anti-cardiac, anti-infective and gastrointestinal medicines at least half the price of branded medicines. While the implementation of this relaunch is pending, this paper analyses a fundamental premise of this scheme ie, generic medicines given through JAS will be cheaper than branded medicines available in the market. A comparative cost analysis of generic medicines available under JAS with branded medicines of the same formulation available in the market was performed.

## Methods

A cost analysis was performed in this study. This study considers the price of the medicine as the direct cost to patients. Various studies have proven that the efficacy and effectiveness of generic medicines is the same as branded medicines.^7,8^ Hence, the price of medicines listed in JAS was compared with the price of the same branded formulation sold in the market. Also, the study assumes that the direct non-medical costs like transportation costs to avail these medicines would be the same for patients accessing either the Jan Aushadhi stores or the private chemists who sell the branded medicines. The sources of cost data include the JAS website and MedGuideIndia.com. Three medicines were selected for comparison. The selection of the three drugs was based on issues of immediate concern expressed by policy-makers and included the following:

1. Medicine of choice for addressing risk factor (cholesterol) for non-communicable disease (NCD): atorvastatin.
2. Commonly prescribed antibiotic in India for serious infectious diseases: cefuroxime axetil.
3. Medicine of choice for anxiety in India: alprazolam.

## Limitations

Since, the study restricts itself to analyzing the direct cost of medicines, no conclusions can be made on the effect on the indirect costs associated with any of these medicines. This study examined only three medications, as the objective was a quick assessment of the objectives of JAS. A detailed study with a larger sample of drugs would give more insights along with analysis of utilization information.

## Results

The JAS medicines cover a wide range of communicable diseases, NCDs (cardiovascular diseases, diabetes), mental disorders like anxiety, analgesics, vitamin, iron, folic acid supplements, and tetanus toxoid injections.^9^ As shown in Table 1, there is a huge variation in the prices of atorvastatin in the branded market. Also, the price of the branded atorvastatin is 2 to 25 times more than the JAS price. Hence, a patient taking statins from the Jan Aushadhi generic drug stores would save substantially. However, as shown in Table 2, in the case of the antibiotic cefuroxime axetil, there are cheaper varieties available in the branded market than the JAS price. However, the price of alprazolam in the JAS is the among the lowest as compared to market prices (Table 3).

**Table 1:** Cost Comparison of Atorvastatin

** Statins**	** Package Unit**	**JAS Price (INR)**	**Branded Price (INR)**		**% Difference With JAS Price**	
			**Lowest**	**Highest**	**Lowest**	**Highest**
Atorvastatin (10 mg)	10 tabs	4.76	12	118.5	152	2389
Atorvastatin (20 mg)	10 tabs	8.56	19	212.38	122	2381

Abbreviations: INR, Indian Rupees; JAS, Jan Aushadhi Scheme.

**Table 2:** Cost Comparison of Cefuroxime Axetil

** Antibiotic**	** Package Unit**	**JAS Price (INR)**	**Branded Price (INR)**		**% Difference With JAS Price**	
			**Lowest**	**Highest**	**Lowest**	**Highest**
Cefuroxime axetil (250 mg)	10 tabs	66.37	26	500	-60	653
Cefuroxime Aaxetil (500 mg)	10 tabs	128.86	43	780	-66	505

Abbreviations: INR, Indian Rupees; JAS, Jan Aushadhi Scheme.

**Table 3:** Cost Comparison of Alprazolam

**Name of drug**	** Package Unit**	**JAS Price (INR)**	**Branded Price (INR)**		**% Difference With JAS Price**	
			**Lowest**	**Highest**	**Lowest**	**Highest**
Alprazolam 0.25 mg	10 tabs	1.84	1	22	-45	1095
Alprazolam 0.50 mg	10 tabs	2.97	6.15	51	107	1617

Abbreviations: INR, Indian Rupees; JAS, Jan Aushadhi Scheme.

There are 256 brands of atorvastatin (10 mg) available in the Indian market and the mean branded price of atorvastatin (10 mg) is INR 57.58 (standard deviation [SD] = 17.90). The difference between JAS price and mean branded price of atorvastatin (10 mg) is INR 52.82. There are 173 brands of atorvastatins (20 mg) available with a mean cost of INR 106.00 (SD = 36.71). The difference between the JAS price and mean branded price of atorvastatin (20 mg) is INR 97.44.

The mean branded price of the 192 available brands of cefuroxime axetil (250 mg) is INR 249.27 (SD = 73.54). The difference between the JAS price and mean branded price of cefuroxime axetil (250 mg ) is INR -182.90. The mean branded price of 180 available brands of cefuroxime axetil (500) is INR 447.23 (SD = 146.09). The difference between the JAS price and mean branded price of cefuroxime axetil (500 mg) is INR -318.37.

The mean branded price of 229 brands of alprazolam (0.25 mg ) is INR 9.35 (SD = 3.09). The difference between JAS price and mean branded price of alprazolam (0.25 mg) is INR 7.51.

The mean branded price of the 244 available brands of alprazolam (0.50 mg) is INR 17.20 (SD = 5.61). The difference between JAS price and mean branded price of alprazolam (0.50 mg) is INR 14.23.

## Discussion

Medicines play a major role in protecting, maintaining and restoring people’s health. The provision of appropriate medicines of assured quality, in adequate quantities and at reasonable prices is, therefore, a concern of global and national policy-makers and agencies implementing health activities and programs.^10^ Drugs and medicines form a substantial portion of OOP spending on health among households in India.^11^ In the context of providing universal healthcare, providing financial risk protection to households affected by illness is a key objective with focus on providing cover for medicines.^12^ In fact, improving access to essential medicines in India is a public health and ethical imperative.^4^

Since medicines constitute the majority of OOP expenses in India, making cheap generic medicines available for patient households would be an important strategy to achieve the objective. In Europe, generic medicines reduced the region’s medicine bill by 61% in 2014.^13^ Even in developing countries, substantial savings could be achieved by switching private sector purchases from branded medicines to lowest-priced generic equivalents.^14^

The JAS is a policy initiative in that direction. The recent union budget of India in 2016 announced an impetus to this scheme.^15^ However, as the study shows, the price of some generic medicines available under JAS is costlier than the corresponding branded medicine available in the market. Furthermore, these are routinely prescribed medicines for common health conditions in the general population. This suggests that the existing pricing of JAS would actually increase the OOP expenditure of patient households, which fails the purpose and objective of the scheme. From the policy perspective, it raises serious questions regarding the pricing of medicines in JAS and the goal to be achieved. From the public perspective, JAS is an important initiative of the Government of India and targets a wide range of medical conditions commonly seen in the population. In India, patients depend heavily on doctors for medicine prescriptions and prescription practices are very poorly regulated. The unethical promotional practices adopted by the pharmaceutical companies make essential medicines unaffordable to the common man.^16^ Also, patients have poor or no knowledge of the price variations among branded and generic medicines, and leave the choice of the medicine to the doctor. Hence, even if there is a regulation for doctors to prescribe only generic medicines from the JAS, it may still not be the cheapest alternative available and would still create a large OOP expenditure on medicines by households. No independent studies have compared the price difference between JAS price and market price of medicine. The only study done on the JAS addressed the issue of quality of generic medicines in the JAS and found that the medicines tested after procuring from Jan Aushadhi sources are of equivalent and comparable quality to their counterpart branded medicines available in the market.^17^ Hence, it is not quality but cost of medicines in JAS which may make the scheme a failure.

## Conclusion

The study shows that while the JAS price is among the lowest in the market for medicines like alprazolam and atorvastatin, there are cheaper branded medicines available in the market for a commonly prescribed antibiotic (cefuroxime axetil). From the policy perspective, it raises serious questions regarding the pricing of medicines in JAS and the goal to be achieved. With information asymmetry and supplier induced demand feature in the healthcare market, the OOP expenditure due to medicines is unlikely to decline in India with the existing JAS. Hence, it is strongly recommended that the Government of India reviews the medicine pricing policy under the JAS. In addition, strong supply side regulation, such as prescription audits, are necessary to prevent the widespread prescription of costly branded medicines. If this is not done, the JAS policy will not be able to meet its objective of providing low-cost affordable medicine and financial risk protection to households from the cost of medicines.

## Ethical issues

Not applicable.

## Competing interests

Author declares that he has no competing interests.

## Author’s contribution

KM is the single author of the paper.
